# Construction and characterization of immunoliposomes targeting fibroblast growth factor receptor 3

**DOI:** 10.1186/s13568-019-0875-5

**Published:** 2019-09-18

**Authors:** Zhong Zheng, Haotian Ji, Wenbo Zong, Qiuju Ran, Xinxin Wang, Xi Yang, Zhuo Zhao, Chengjun Yang, Yechen Xiao

**Affiliations:** 1 Department of Biochemistry and Molecular Biology, College of Basic Medical Science, Jilin University, Changchun, 130021 China; 2 School of Life Science, Jilin Normal University, Siping, 136000 China

**Keywords:** Fibroblast growth factor receptor 3, Single chain variable fragment, Liposomes, Immunoliposomes

## Abstract

Fibroblast growth factor receptor 3 (FGFR3) plays an important regulatory role in tumor cell proliferation and drug resistance. FGFR3 is often constitutively active in many tumors. To deliver drugs into tumor cells by targeting FGFR3 will be a promising and potential strategy for cancer therapy. In this study, a novel fusion protein, ScFv-Cys containing a single chain variable fragment (ScFv) and an additional C-terminal cysteine residue, was generated at a rate of 10 mg/L of bacterial culture and purified at 95% by Ni-NTA chromatography. Subsequently, the recombinant ScFv-Cys was coupled with malPEG2000-DSPE and incorporated into liposomes to generate the immunoliposomes. The results indicated that immunoliposomes can specifically deliver the fluorescent molecules, Dio into bladder cancer cells highly expressing FGFR3. In conclusion, we successfully generated FGFR3-specific immunoliposomes, and proved its targeting effect and delivering ability.

## Introduction

Fibroblast growth factor receptors (FGFRs) are glycoproteins comprising of two or three Ig-like extracellular domains, a hydrophobic transmembrane domain, and a cytoplasmic region that contains a tyrosine kinase domain (Wesche et al. 2011). Binding of fibroblast growth factor (FGF) ligands promotes receptor dimerization and activation of the tyrosine kinase domain, resulting in autophosphorylation of the intracellular kinase domain and a downstream activation of intracellular signaling cascades (Wesche et al. 2011). FGFs and their receptors regulate a multitude of cellular process, including cell proliferation, differentiation, migration, and survival during embryo development and adult stage (Beenken and Mohammadi 2009).

Among four FGFR members, FGFR3 is mainly expressed during embryogenesis and development of bone, brain, ear, and urothelial organ (Wesche et al. 2011). Dysfunctional expression of FGFR3 or FGFR3 mutations were highly linked to many types of cancer, such as multiple myeloma (MM) (Qing et al. 2009), bladder cancer (Lott et al. 2009; Qing et al. 2009), breast cancer (Wang and Ding 2017) and colorectal cancer (Fromme et al. 2018), etc. Activated FGFR3 signaling pathway has been indicated to promote tumor growth, metastasis, and resistance to drug (Natalia et al. 2018; Saichaemchan et al. 2016). Therefore, it is no surprise to aim FGFR3 as potential therapeutic target for understanding the disease development and cancer therapy.

To date, there are some strategies to inhibit FGFR3 functions, such as small molecular inhibitor, small interfering RNA (siRNA), monoclonal antibody and single chain variable fragment (ScFv), etc. Several small molecular inhibitors against FGFR3 tyrosine kinase have shown cytotoxic responses and anti-cancer effects in the bladder cancer and MM (Saichaemchan et al. 2016). However, most of small molecule inhibitors against FGFR3 lack specificity and have a similar inhibitory effect on other FGFRs or VEGF as well as c-kit (Patani et al. 2016). Thus, it generates many side effects in vivo and also limits its clinical application. siRNA is unstable in vivo and easier to be degraded (Singh et al. 2018), so there are no good methods to overcome this difficulty now. In contrast, the use of monoclonal antibodies or ScFv, which have strong antigen binding affinity and high specificity, presents an attractive strategy. ScFv additionally has the characteristics of low molecular weight, short half-life in blood and weak immunogenicity (Ahmad et al. 2012; Fercher et al. 2018). Therefore, ScFv against FGFR3 has been screened and applied to study antitumor activity in bladder cancer for a long time (Martinez-Torrecuadrada et al. 2005, 2008).

In recent years, combination of ScFv-mediated target therapy and siRNA-induced gene therapy has become a potential therapeutic approach in tumor therapy. A novel type of fusion protein containing ScFv and basic polypeptide (e.g., protamine, 9-arginine) was created to deliver siRNA into ScFv-targeted cancer cells. For example, fusion protein ScFv-9R, which has previously been designed by our lab, contains ScFv against FGFR3 and 9-arginine. ScFv-9R can both bind siRNA and deliver siRNA into FGFR3 positive cancer cells via silencing specific gene expression to suppress tumor growth (Zhang et al. 2014). Afterwards, we created another fusion protein, R3P, which is composed of ScFv and protamine (Zang et al. 2017). R3P, like ScFv-9R, can deliver siRNA into FGFR3 positive cancer cells. Although ScFv-9R and R3P both had good targeting effect and delivering function, nude siRNA could not be protected in the complex of siRNA and fusion protein so that it is easier to be degraded by RNase in vivo. In addition, ScFv-9R or R3P cannot deliver chemotherapy drugs. These shortcomings all limit their applications.

To meet the need of both chemotherapy drug delivery and target therapy of cancer, immunoliposomes emerge at the right moment. They are composed of modified liposome and ScFv. Liposome is a single layer or multilayer vesicle composed of lipid bilayer, and can be absorbed by the reticuloendothelial system after intravenous injection, so it is widely used in the transportation of anticancer drugs (Feng et al. 2017). Immunoliposomes can recognize specific receptors of tumor cells and deliver drugs or siRNA into the target cells (Ju et al. 2014; Niwa et al. 2018). Immunoliposomes have been used as targeted carriers for drugs, such as pancreatic ductal adenocarcinoma (Urey et al. 2017), breast cancer (Rodallec et al. 2018) and skin cancer (Petrilli et al. 2018).

In this study, we first expressed and purified ScFv-Cys containing the FGFR3-specific ScFv and an additional C-terminal cysteine residue in recombinant *E. coli*, then prepared ScFv-PEG2000-DSPE by conjugating malPEG2000-DSPE with ScFv-Cys, followed by coupling with liposomes. Both the liposome targeting modification and the long-term liposome modification by PEG2000 were completed. Our results showed that FGFR3-specific immunoliposomes have better delivery efficiency for chemotherapy drugs or siRNA and will be a promising targeting therapeutic vehicle.

## Materials and methods

### Materials

Prime STAR^®^GXL DNA Polymerase was purchased from TaKaRa Company (Japan). Restriction enzymes *Nde*I and *Xho*I were purchased from NEB (England). RNeasy Mini Kit was purchased from QIAGEN (Gemany). The SuperScript^®^ III First-Strand Synthesis System was purchased from Invitrogen (USA). SYBR Green kit was purchased from TransGen Biotech (China). Gel extraction kit and plasmid miniprep kit were obtained from the CW Bio Company (China). Ni-NTA agarose were purchased from GE Healthcare (Sweden). Pierce^®^ BCA Protein Assay Kit was purchased from Thermo Company (USA). Cholesterol, hydrogenated soy phosphatidylcholine (HSPC), mPEG2000-DSPE and mal-PEG2000-DSPE were purchased from Avanti polar lipids (USA). Hoechst 33342 and Dio were purchased from Sigma (USA). The human bladder cancer cell line RT112 and T24 were supported by JENNIO Company (China).

### Construction of pET-20b-ScFv-Cys expression vector

The PCR primers were designed according to the ScFv sequence (Liu et al. 2015) from GenBank (accession number KP405837), and synthesized by the Sangon Biotech Company (Shanghai). The forward primer (F1) was designed as followed (51nt): 5′-GGAATTCCATATGCATCATCATCATCATC ACGAAGTGCAGCTGCTGGAAAG-3′ according to the 5′ terminal sequence of ScFv. The reverse primer (R1) that contained 3′ terminal sequence of ScFv, linker and Cysteine (Cys) was synthesized as followed (67 nt): 5′-CGCTCGAGTTAGCAACCGCTACCGCTGCTACCACCGCTGCTACCACCG CGTTTAATTTCCACTTTGG-3′. The restriction endonuclease *Nde*I and *Xho*I sites were added in the forward and reverse primer, respectively. The target gene was obtained by PCR amplification using F1 and R1 as the forward and reverse primers, using the ScFv gene as the template (Liu et al. 2015). PCR parameters consisted of 5 min of pre-denaturation activation at 98 °C, followed by 30 cycles of denaturation at 98 °C for 10 s, annealing at 55 °C for 30 s, extension at 72 °C for 60 s, and then a final single extension at 68 °C for 5 min. The PCR product and the pET-20b empty vector were digested with *Nde*I and *Xho*I, and ligated using T4 DNA ligase, then the ligated plasmid was transformed into *E. coli* DH5α competent cells. Finally, the recombinant plasmid was verified by double enzyme digestion and confirmed by DNA sequencing (Sangon, Shanghai).

### Inducible expression of recombinant ScFv-Cys

The recombinant plasmid was transformed into *E. coli* BL21(DE3), and several colonies were selected to induce the recombinant protein expression in a 4 mL fresh Luria–Bertani (LB) medium (1% peptone, 0.5% yeast extract, and 1% sodium chloride, pH 7.0), and cultured in a shaker at 37 °C. When the OD_600_ attained 0.6 to 0.8, 0.5 mM isopropyl-d-thiogalactoside (IPTG) was added into bacteria medium for 4 h induction at 37 °C along with shaking of 200 rpm. The expression of recombinant ScFv-Cys (rScFv-Cys) was detected by SDS-PAGE, and the bacteria clone with the highest expression level was used to induce the expression in a large scale. The detailed methods have been reported previously (Zhang et al. 2014).

### Purification and identification of rScFv-Cys

rScFv-Cys was purified by Ni-NTA chromatography, and the detailed method was consistent with the previous report (Liu et al. 2017). After affinity purification employing Ni-column, rScFv-Cys was desalinated with 3 kDa dialysate bag, and concentrated using an ultrafiltration tube. The protein concentration was determined by BCA protein quantitative assay kit (Thermo, USA), and its purity was analyzed by SDS-PAGE. The target protein was cut off from gel and placed in a 1.5 mL tube containing ddH_2_O, and was identified by liquid chromatography–tandem mass spectrometry (LC–MS/MS) from Huada Protein Co. Ltd. (Beijing).

### Preparation and characterization of liposomes and immunoliposomes

Liposomes were prepared by thin-film dispersion method. In brief, HSPC, cholesterol and mPEG2000-DSPE (20:10:1, molar ratio) were dissolved in chloroform. The chloroform was evaporated slowly at 42 °C by rotary evaporator. After the chloroform was completely dried, 2-hydroxyethyl (HEPES) at the concentration of 10 mM was added into this mixture and performed to ultrasonication for 20 min at 20 °C, then liposome solution was filtered by filter member of 450 nm and 220 nm, and the different particle size of liposomes was produced.

To further synthesize ScFv-PEG2000-DSPE, adding DTT to the previously purified rScFv-Cys protein to final concentration 50 mM at 4 °C for 1 h reaction, the thiol group of cysteine residue of rScFv-Cys was fully recovered. The reduced ScFv-Cys was added to the dialysis bag. The extra DTT was removed by 20 mM Tris–HCl and dialyzed at 4 °C for 10 h. Before dialysis, sufficient nitrogen was added to the dialysate to insulate the oxygen for preventing the single chain antibody from oxidation.

ScFv-Cys and malPEG2000-DSPE were mixed in the ratio of 1:4, and was gently shaken at room temperature for 16 h to ensure the full coupling of two components. Also, before the reaction, full nitrogen protection was applied. The target substance ScFv-PEG2000-DSPE was obtained by this method. The particle size of liposomes and immunoliposomes was measured by Marvin laser particle size analyzer.

### RNA isolation, cDNA synthesis and real-time RT-PCR

Total RNA of RT112 and T24 cells was extracted using the RNeasy Mini Kit (QIAGEN), and reversely transcribed into cDNA using reverse transcription (RT) PCR kit (The SuperScript^®^ III First-Strand Synthesis System, Invitrogen). PCR was performed with SYBR Green kit (TransGen Biotech, China) by ABI Prism 7000. The two pair of primers against different regions of FGFR3 were designed and named as FGFR3-1 and FGFR3-2. The sequences of forward and reverse prime of FGFR3-1 are 5′- GGGAGGACGAGGCTGAGGAC-3′ and 5′-GATGGAGGGAGTGGGGTTGC-3′, respectively. The sequences for forward and reverse prime of FGFR3-2 are 5′-CCCACTCCCTCCATCTCCTG-3′ and 5′-GCTGCCAAACTTGTTCTCCA-3′, respectively. Gapdh was used as the internal control, and its forward and reverse prime sequences are 5′-TGCACCACCAACTGCTTAGC-3′ and 5′-GGCATG GACTGTGGTCATGA-3′, respectively. PCR parameters consisted of 5 min of DNA Polymerase activation at 94 °C, followed by 40 cycles of denaturation at 94 °C for 30 s, annealing at 55 °C for 30 s. After the reaction, ABI Prism 7000 software was used to statistically analyze the difference of gene transcription between different groups.

### The targeted effect and delivery ability of immunoliposomes

200,000 RT112 cells and T24 cells were cultured in DMEM medium, with 10% FBS at 37 °C in the cell incubator, respectively. The fluorescence-labeled liposomes and immunoliposomes were prepared by mixing 0.1% Dio, and added into cells with 800 μl cell medium for 3 h in the dark. Cell medium was removed, and washed three times with PBS buffer. Hoechst 33342 was added to a final concentration of 5 μg/mL for 10 min in the dark. The staining solution was then removed, and cells were washed three times with PBS, and the medium was replaced for DMEM complete medium. Cells were observed by fluorescent microscopy (Olympus, Japan).

## Results

### Construction of pET-20b-ScFv-Cys expression vector

**Fig. 1:**
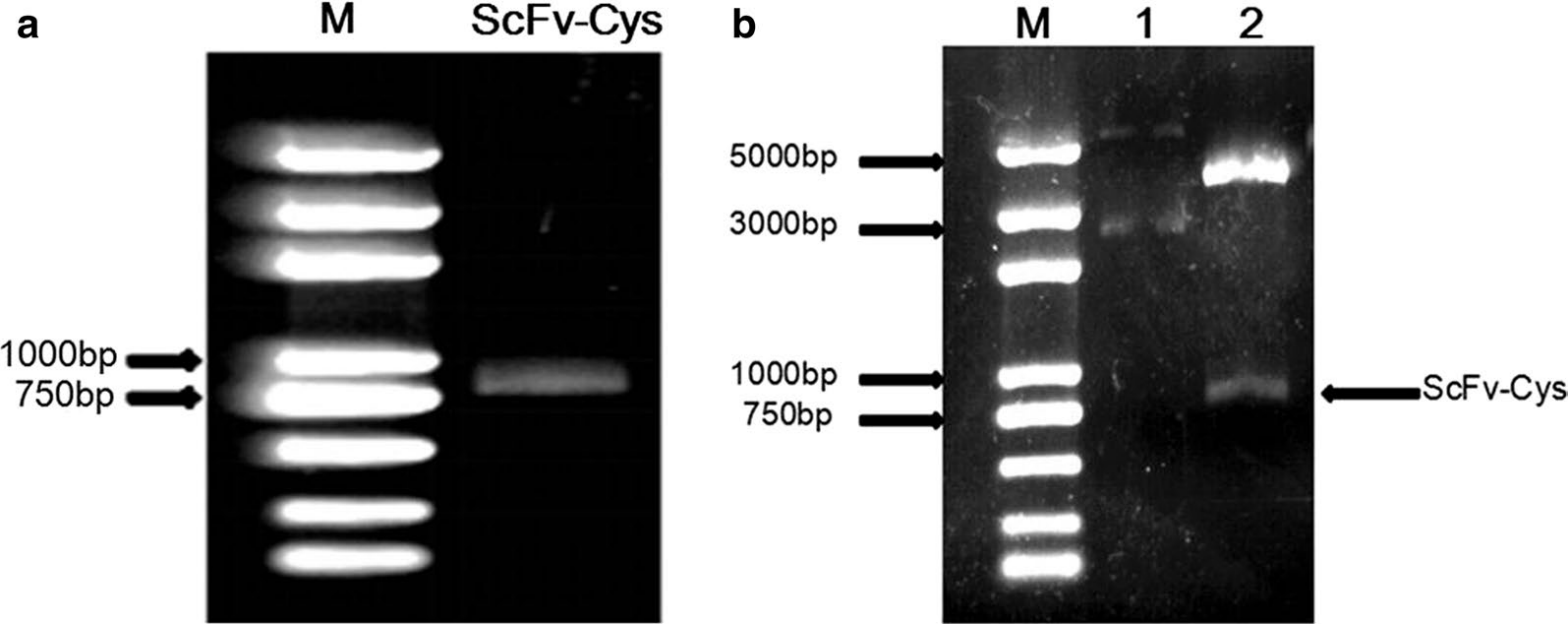
Synthesis of ScFv-Cys and construction of recombinant plasmid. **a** The PCR product of ScFv-Cys. **b** Identification of the recombinant plasmid by enzyme digestion. Lane 1, the recombinant plasmid without enzyme digestion. Lane 2, the digested recombinant plasmid with *Nde*I and *Xho*I

According to the strategy that was described in “Materials and methods”, the ScFv-Cys gene was synthesized by PCR amplification. As shown in Fig. 1a, the PCR product of ScFv-Cys gene was analyzed by 1% agarose gel electrophoresis, and its size is about 760 bp. The final PCR product was digested by two restriction enzymes (*Nde*I and *Xho*I), and cloned into the expression vector pET-20b. The results showed that the insert fragment can be cut in the recombinant plasmid by double enzyme digestion (Fig. 1b), and further confirmed the sequence of rScFv-Cys is in conformity with the expected sequence by automated DNA sequencing (data not shown).

### Induced expression and purification of rScFv-Cys

**Fig. 2:**
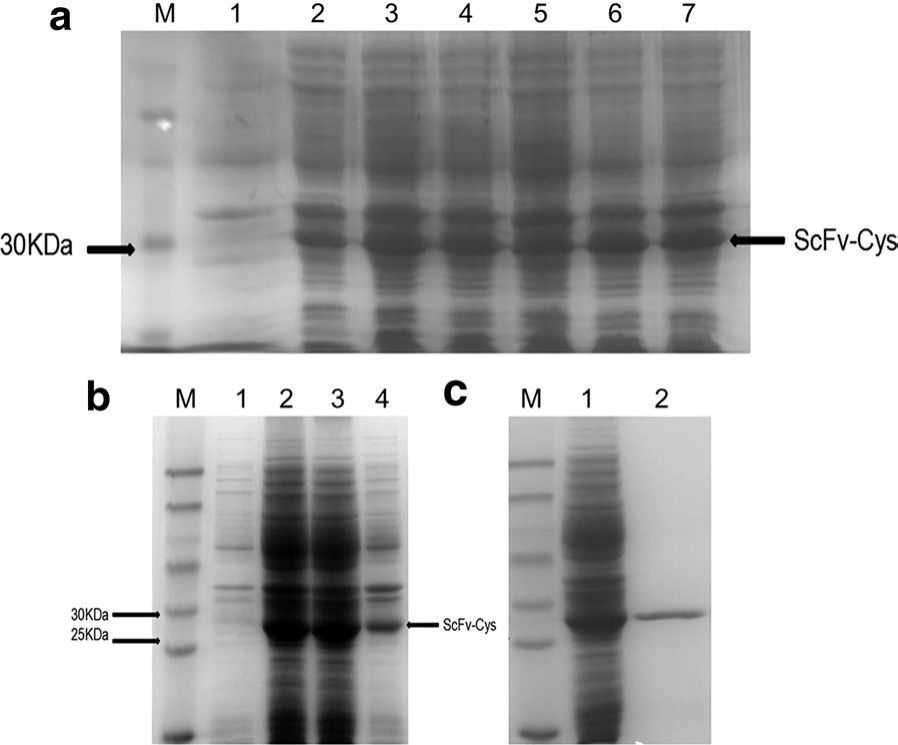
Inducible expression and purification of rScFv-Cys. **a** SDS-PAGE analysis of rScFv-Cys. M, Marker. Lane 1, total bacterial protein without IPTG. Lane 2–7, total bacterial protein with IPTG induction. **b** SDS-PAGE analysis of different expression levels of rScFv-Cys in the supernatant and precipitation of bacterial. Lane 1, total bacterial protein without IPTG. Lane 2, total bacterial protein after induction. Lane 3, bacterial supernatant after induction; Lane 4, bacterial precipitation after induction. **c** SDS-PAGE analysis of rScFv-Cys purification (lane 1, before purification; lane 2, after purification)

Recombinants were inoculated in fresh LB medium, and incubated in a shaking incubator at 37 °C until the OD_600_ was 0.6 to 0.8. IPTG was then added to a final concentration of 0.5 mM for induced expression of recombinants. After 16 h induction, the bacteria were harvested by centrifugation, and protein was extracted and separated by sonication and centrifugation. The supernatants and pellets of bacteria were collected and subjected to 12% SDS-PAGE analysis. As shown in Fig. 2a, inducible protein was produced in abundance in IPTG-treated recombinant bacterial compared to control, and its molecular weight is about 26 kDa, which corresponds to the predicted size of rScFv-Cys. The target protein is more than 50% of total bacterial protein and the soluble fraction reaches 80% of total expressed recombinant protein (Fig. 2b).

Ni-NTA affinity column was chosen for purification due to the introduced 6-His tag in the 5′ terminal of ScFv-Cys gene. rScFv-Cys protein was eluted from Ni-NTA column using Tris–HCl containing 200 mM imidazole. SDS-PAGE analysis showed that the purity of rScFv-Cys was more than 95% (Fig. 2c).

### Identification of rScFv-Cys

**Fig. 3:**
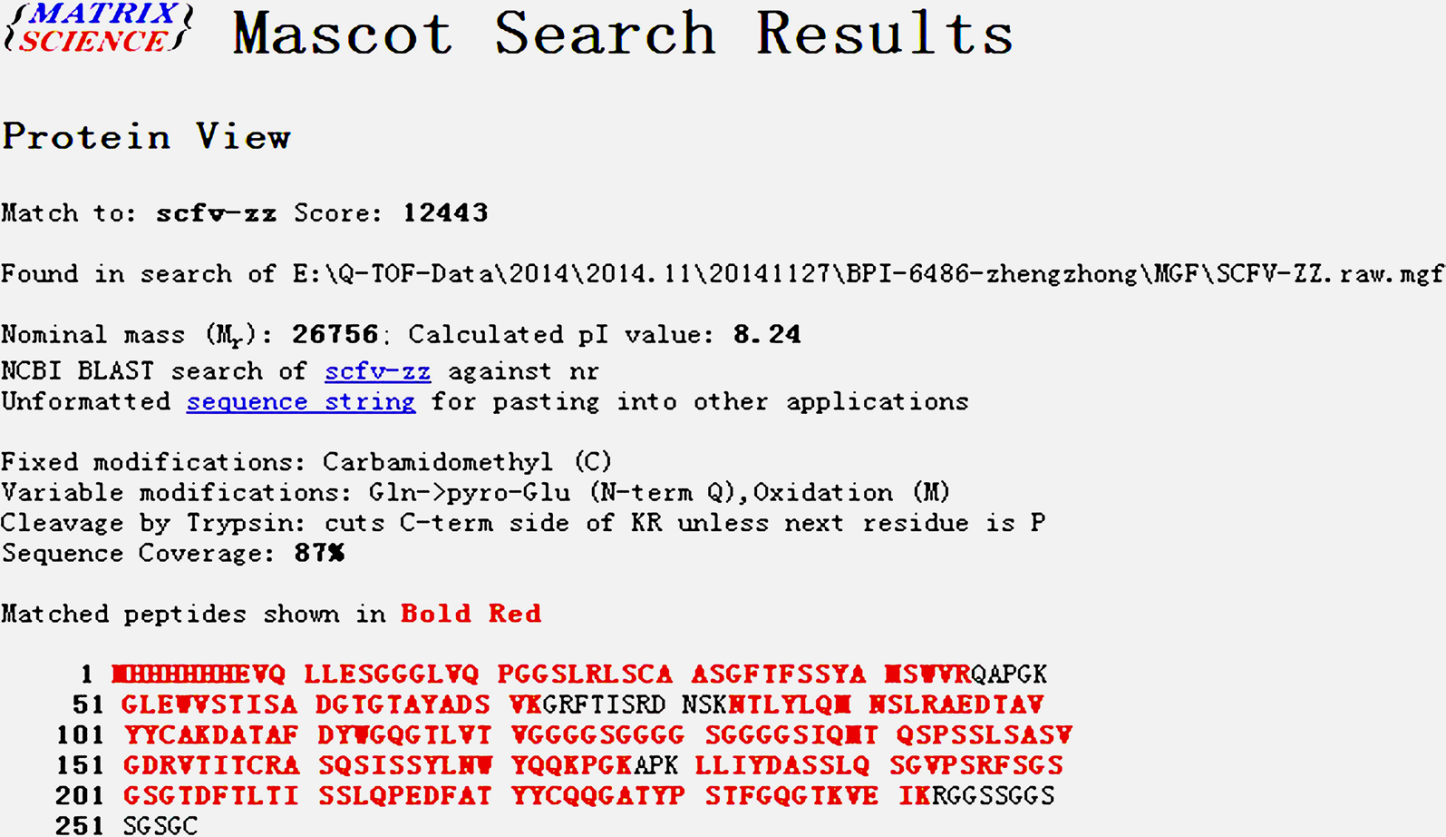
Identification of rScFv-Cys. The purified rScFv-Cys was assayed by Mass spectrometric method. The peptides in bold red highly match with rScFv-Cys, in which its sequence coverage reaches 87%

To verify the authenticity of amino acid sequence, the rScFv-Cys was evaluated by mass spectrometric analysis (LC–MS/MS). When the analysis coverage is more than 50%, the score of higher than 200 indicated that the protein sequence is highly correct. As shown in Fig. 3, the analysis score of rScFv-Cys is 12443, the sequence coverage is 87% and nominal mass is 26,756 Da. These results demonstrated that rScFv-Cys was consistent with the expected result.

### Synthesis of DSPE-PEG2000-ScFv

**Fig. 4:**
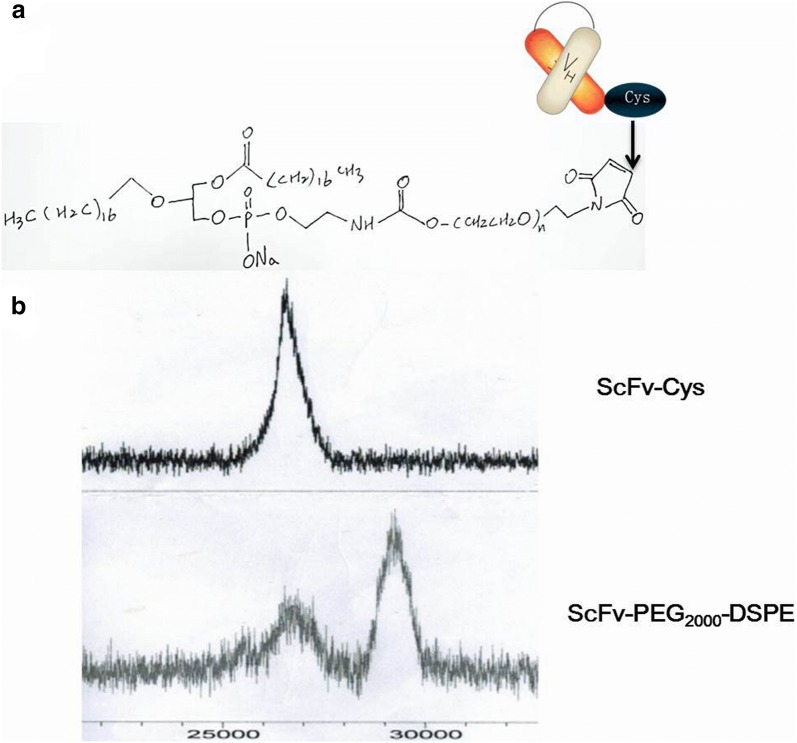
Analysis of ScFv-PEG2000-DSPE by MALDI-TOF–MS. **a** The coupling schematic of PEG2000-DSPE and ScFv-Cys. **b** The purified product of DSPE-PEG2000-ScFv was verified by MALDI-TOF MS. The theoretical molecular weight (MW) of malDSPE-PEG2000 is 3 kDa, so the experimental MW of ScFv and DSPE-PEG2000-ScFv were exactly 26 kDa and 29 kDa, respectively

Purified rScFv-Cys was coupled with DSPE-PEG2000 according to the schematic design (Fig. 4a), and methods were described in “Materials and methods”. The purified product of DSPE-PEG2000-ScFv was verified by matrix-assisted laser desorption/ionization time of flight mass spectrometry (MALDI-TOF–MS). The experimental molecular weight (MW) of ScFv and DSPE-PEG2000-ScFv were 26 kDa and 29 kDa (Fig. 4b), respectively. The increased 3 kDa in DSPE-PEG2000-ScFv complex was just the theoretical MW of malDSPE-PEG2000, suggesting that DSPE-PEG2000-ScFv has been successfully synthesized.

### Preparation and characteristics of liposomes and immunoliposomes

**Fig. 5:**
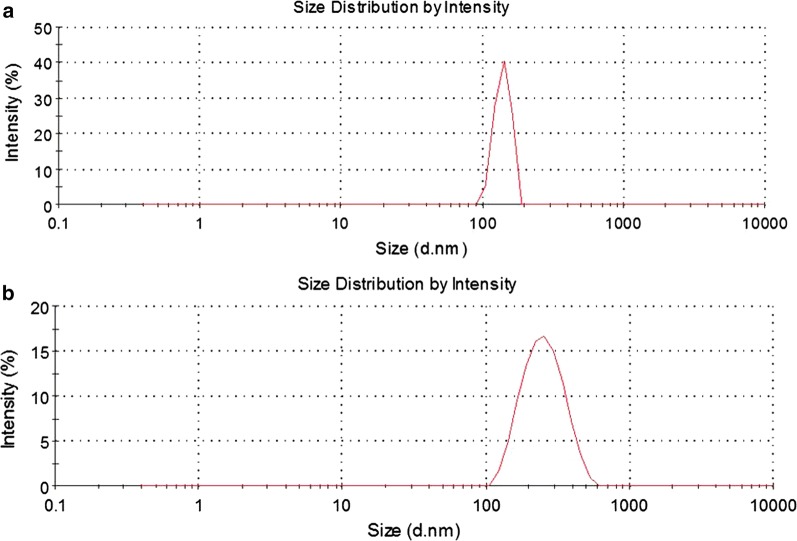
Characteristics of liposomes and immunoliposomes. The mean particle sizes of liposomes and immunoliposomes were measured by Malvern Zetasizer Nano ZS90 instrument (Malvern Instruments Ltd., UK). **a** Shows the size of liposomes, and **b** shows the size of immunoliposomes

The mean particle size of liposomes and immunoliposomes was measured by Malvern Zetasizer Nano ZS90 instrument (Malvern Instruments Ltd., UK). The calculation parameters consist of intensity, number, volume and statistics graph. As shown in Fig. 5, the particle size of liposomes and immunoliposomes were 128.3 nm and 189.1 nm, respectively. The polydispersity (DPI) of liposomes and immunoliposomes were both less than 0.3, which fits to the stability requirement (0.183 and 0.209, respectively). The correct particle size and spheroid morphology indicated that liposomes and immunoliposomes have been successfully synthesized.

### In vitro cellular targeting assay

**Fig. 6:**
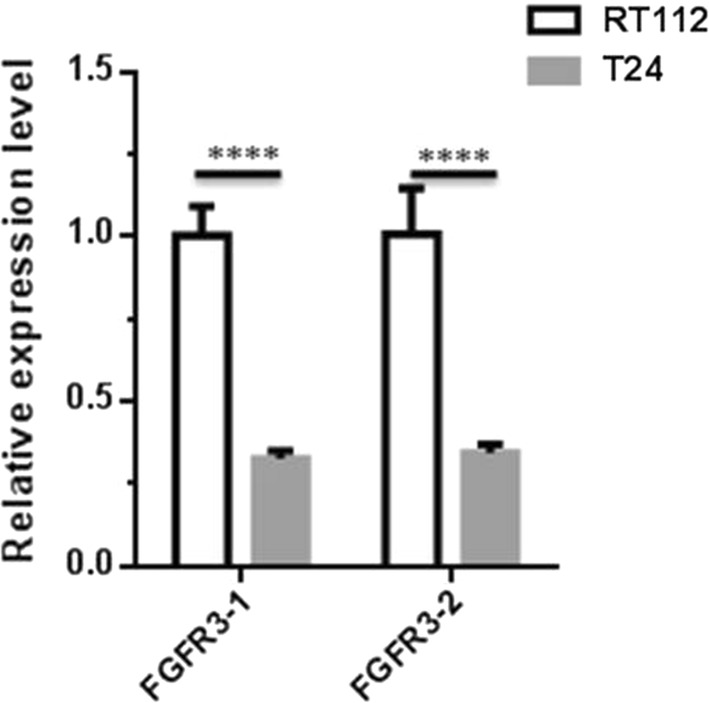
The expression level of FGFR3 in RT112 and T24 cells. The total RNA of RT112 and T24 cells was extracted, respectively. The reverse transcription was performed according to the kit protocol, and PCR was done with two pair of primers against different regions of FGFR3 according to the descriptions of “Materials and methods”. The result showed that FGFR3 both expressed in RT112 and T24 cells, but the expression level of FGFR3 in RT112 cells is threefold higher than that of in T24 cells. *P *< 0.0001, n = 4

**Fig. 7:**
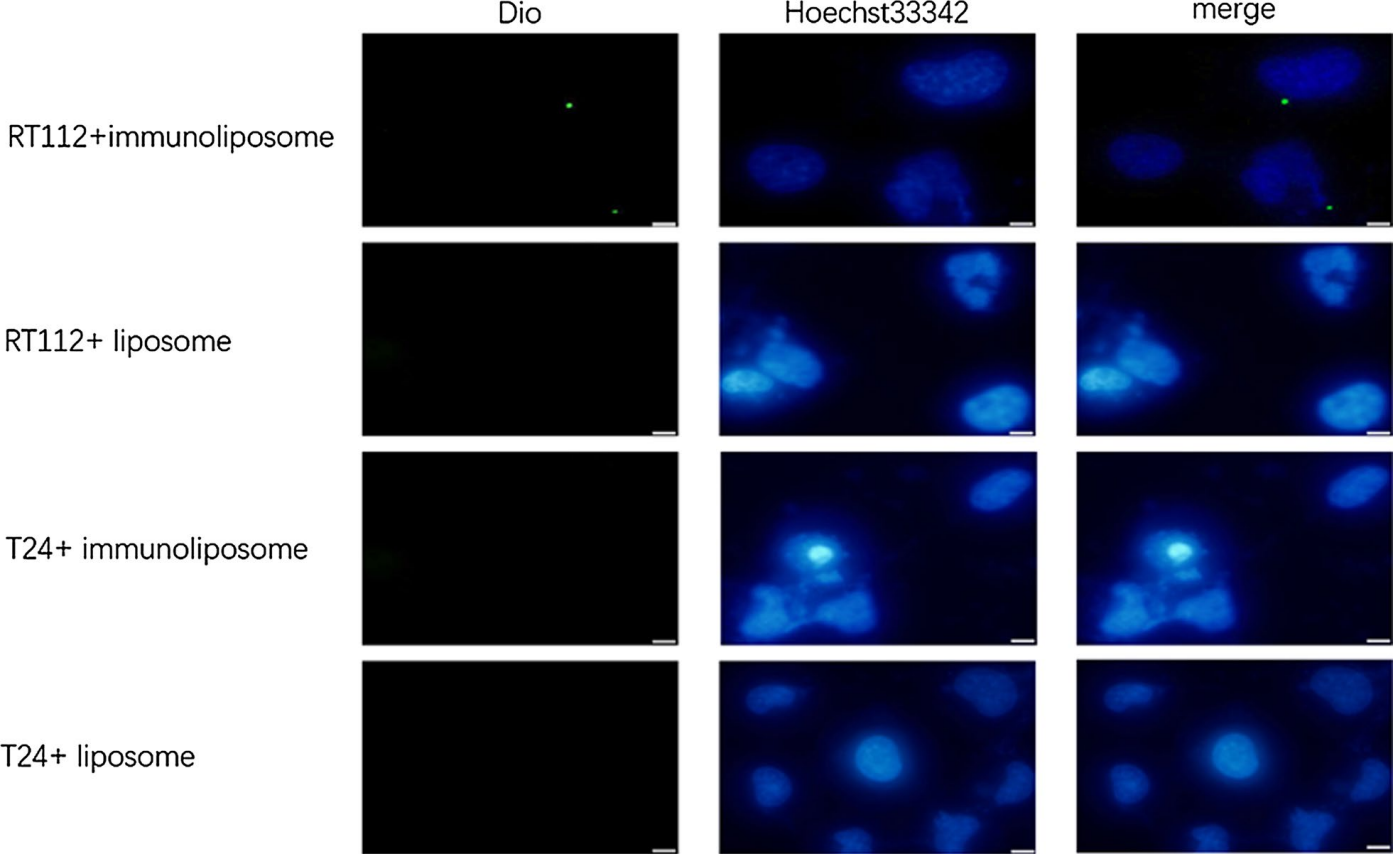
Dio delivered by ScFv-PEG2000-DSPE into RT112 cells in vitro. The immunoliposomes or liposomes containing 0.1% Dio were incubated with RT112 cells and T24 cells in cell incubator for 3 h in the dark. Hoechst 33342 was used to label cell nuclear. The left panel displayed the green Dio in different treatment groups, the middle panel showed the blue cell nuclear stained by hoechst 33342, and the right panel showed merged figures of two colors. The cells were observed under a fluorescent microcopy. Bar = 50 μM

To estimate the targeting and delivering efficiency of immunoliposomes, the two bladder cancer cell lines, RT112 and T24 were chosen as target cells. The real-time RT-PCR was performed with two pair of primers against different regions of FGFR3, and the results both displayed that the expression level of FGFR3 in RT112 cells is threefold higher than that in T24 cells (Fig. 6), so RT112 and T24 cells in our studies were considered as high FGFR3-expressing and low FGFR3-expressing cell lines, respectively. Dio, a fluorescent lipophilic tracer, was used to label immunoliposomes or liposomes, and Hoechst 33342 was used to stain cell nuclear. As shown in Fig. 7, more green fluorescence beside the nuclear staining (blue color) was observed in immunoliposomes-treated RT112 cells compared with that of the liposome control group, suggesting immunoliposomes are more effective to deliver Dio into high FGFR3-expressing RT112 cells. In contrast, less green fluorescence was found in T24 cells treated by either liposomes or immunoliposomes. These results indicate that immunoliposomes can play a better targeting role towards high FGFR3-expressing cancer cells compared to liposomes.

## Discussion

Tumorigenesis is a complicated systematic course which probably involves in the multiple genes and many environmental factors. Activation of oncogenes or dysfunctional of tumor suppressor genes is very important cause of tumorigenesis in most of cancer types. Overexpression or active mutation of FGFR3 has been found in many types of cancers, such as bladder cancer, MM, etc. So to set up a deliver system by targeting FGFR3 will be very promising in clinical cancer therapy. Our previous study introduced a novel delivery tool, R3P, which is composed of ScFv against FGFR3 and protamine. R3P can efficiently deliver siRNA into FGFR3-positive tumor cells, and induce cell apoptosis by decreasing the expression of oncogenes. However, siRNA delivered by R3P is easier to be degraded in vivo due to lack of protection. In addition, R3P could not deliver chemotherapy drug. To resolve above-mentioned shortcomings, we generated FGFR3-specific immunoliposomes by using ScFv against FGFR3 (Liu et al. 2015) in this study. In order to achieve ligation between ScFv and liposome, we firstly expressed and purified rScFv-Cys in *E. coli,* which has 10 mg/L yield and more than 95% purity. rScFv-Cys in vitro was treated and generated disulfide bond to link PEGylated liposomes. Then we chose to connect the target material with liposome by the double bond of cysteine residue of ScFv-Cys and mal-PEG2000-DSPE. Because cysteine is easily oxidized, the mercapto group needs be reduced to mal-PEG2000-DSPE before connection with DTT and other substances. It is necessary to remove them by dialysis to avoid interference with the coupling reaction since DTT itself contains thiol groups. The protection by nitrogen gas is very important for the reduction of thiol group, the removal of thiol group and the final coupling process. The failure of the whole coupling reaction is easily caused by the incomplete protection by nitrogen at any one step.

In this study, we observed more green fluorescence in immunoliposomes-treated RT112 compared with that of liposomes group (Fig. 7), suggesting immunoliposomes are more effective to deliver Dio into RT112 cells with high FGFR3 level. However, fluorescence intensity is very poor. One possible reason is that Dio is a kind of weak fluorescent substance for labeling liposome. In the future, this limitation might be overcome by using strong fluorescence substance-labeled siRNA (Song et al. 2016) or chemotherapeutic drugs (Urey et al. 2017). On the other hand, the poor targeting effect may be caused by the low content of ScFv in liposomes, so we need to further optimize the liposome preparation method by increasing the proportion of rScFv.

Although the immunoliposomes targeting FGFR3 have been successfully prepared, there are still many follow-up studies to be done, such as the optimization of preparation process, encapsulation of chemotherapeutic drugs and nucleic acid drugs as well as the reduction of liposome costs, etc.

## Data Availability

Data will be made available through publication.
